# The role of MET in chemotherapy resistance

**DOI:** 10.1038/s41388-020-01577-5

**Published:** 2021-02-01

**Authors:** Georgina E. Wood, Helen Hockings, Danielle M. Hilton, Stéphanie Kermorgant

**Affiliations:** Barts Cancer Institute, Queen Mary University of London, John Vane Science Centre, Charterhouse Square, London, EC1M 6BQ UK

**Keywords:** Cancer models, Cancer therapeutic resistance, Targeted therapies, Growth factor signalling

## Abstract

Chemotherapy remains the mainstay of treatment in the majority of solid and haematological malignancies. Resistance to cytotoxic chemotherapy is a major clinical problem and substantial research is ongoing into potential methods of overcoming this resistance. One major target, the receptor tyrosine kinase MET, has generated increasing interest with multiple clinical trials in progress. Overexpression of MET is frequently observed in a range of different cancers and is associated with poor prognosis. Studies have shown that MET promotes resistance to targeted therapies, including those targeting EGFR, BRAF and MEK. More recently, several reports suggest that MET also contributes to cytotoxic chemotherapy resistance. Here we review the preclinical evidence of MET’s role in chemotherapy resistance, the mechanisms by which this resistance is mediated and the translational relevance of MET inhibitor therapy for patients with chemotherapy resistant disease.

## Introduction

**Fig. 1:**
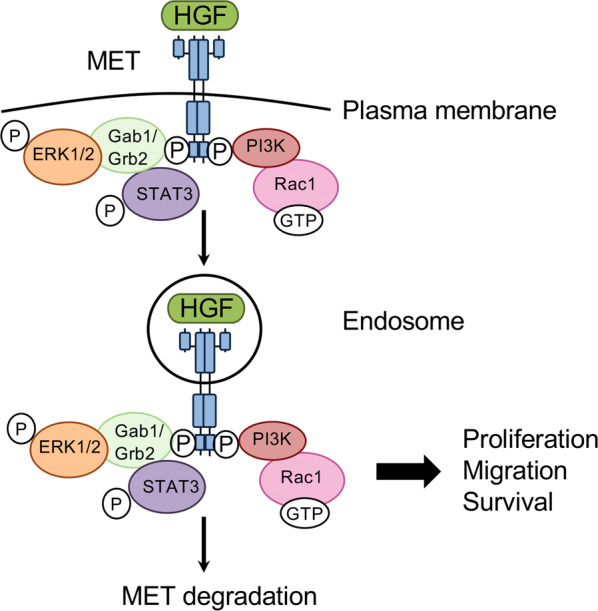
MET signalling. HGF binding to MET triggers MET dimerisation and phosphorylation. Several signalling pathways are then activated, leading to the activation of signals including ERK1/2, STAT3, PI3K and Rac1. Cells respond in increasing their proliferation, migration and/or survival. In parallel, HGF binding to MET triggers its internalisation. MET is still bound to HGF and continues to signal on endosomes. MET internalisation has been shown to be required to sustain its signalling. MET slowly gets degraded, leading to signal termination but oncogenic MET mutants are protected against degradation and trigger persistent endosomal signalling, contributing to their oncogenicity.

The protein MET (also termed Met, c-Met, c-MET), encoded by the proto-oncogene *MET* found on chromosome 7q31, is a cell surface receptor tyrosine kinase (RTK) predominantly expressed by epithelial cells [1, 2]. Upon binding of its only known ligand, hepatocyte growth factor (HGF), MET homodimerises, phosphorylates and triggers the stimulation of a complex system of intracellular signalling cascades. This leads to the activation of key molecules such as extracellular signal-regulated kinase 1 or 2 (ERK1/2), the phosphoinositide 3-kinase–AKT axis (PI3K/AKT), signal transducer and activator of transcription 3 (STAT3) and Rac1 [1]. As a consequence, cells increase their proliferation, survival and/or motility. Interestingly, MET activation by its ligand HGF, also triggers rapid MET endocytosis through a dynamin and clathrin mediated pathway [3]. Although most endocytosed MET is degraded, this occurs at a slow pace. MET remains active and triggers activation of ERK1/2, STAT3 and Rac1 from within endosomes [3–5], as opposed to the classical view of RTK signalling from the plasma membrane only (Fig. 1). Furthermore, oncogenic forms of MET display modified endocytosis and/or ubiquitination leading to enhanced stability, contributing to their malignant potential [6].

The effects of MET activation are crucial to physiological processes such as embryonic development, organ development and wound healing [7]. HGF is a pleiotropic factor produced by mesenchymal cells in the stroma and as such is widely distributed in the extracellular matrix (ECM) of most tissues [1, 8]. Dysregulation of the MET/HGF pathway leads to uncontrolled cell proliferation and oncogenesis and is observed in multiple tumour types [9]. Germline and somatic mutations in MET’s kinase domain leading to constitutive activation are seen in renal papillary carcinoma, childhood hepatocellular carcinoma and colorectal cancer [10–12], whilst intronic mutations leading to an exon 14 deleted splice variant are seen in non-small cell lung cancer (NSCLC) [6]. Increased levels of serum and intra-tumoural HGF are observed in NSCLC and breast cancer [13, 14].

MET amplification/overexpression has been shown to lead to dependence on MET for cell survival [15]. An increasing number of MET/HGF-directed therapies aiming to target this oncogene addiction are being developed. There are three tyrosine kinase inhibitors (TKIs) which target MET that are currently approved for use as monotherapy in the treatment of cancers - crizotinib (PF-02341066), cabozantinib (XL-184) and capmatinib (INC280). Crizotinib and cabozantinib are both multitarget inhibitors, their main targets being ALK, ROS1 and MET for crizotinib, and VEGFRs, AXL, RET and MET for cabozantinib. Capmatinib, a selective MET inhibitor, has recently been FDA-approved for use in advanced NSCLC patients with a *MET* exon 14 mutation following the GEOMETRY mono-1 trial [16]. Table 1 gives an overview of MET/HGF-directed drugs that have been investigated in preclinical and/or clinical research and highlights those that have progressed to phase III trials. Previous reviews have comprehensively summarised details of all of these studies including a recent review of the most relevant or ongoing studies [17].

**Table 1:** A summary of the drugs that have been developed to target the MET/HGF signalling pathway and their use in clinical trials, with a focus on those which have reached Phase III (completed or ongoing).

	Inhibitor name	Phase III ongoing-completed
Anti-HGF monoclonal antibodies	Rilotumumab/AMG-102	NCT02154490
	Ficlatuzumab/AV-299/SCH 900105	Not yet conducted – trials completed and ongoing at Phase 2
	HuL2G7/TAK701	Not yet conducted – trial completed at Phase 1, none ongoing
	YYB-101	Not yet conducted – trials completed at Phase 1 and ongoing at Phase 2
MET antagonists	Onartuzumab/RO5490258/PRO-142966	NCT02488330, NCT01662869, NCT01887886, NCT02031744
	SAIT301	Not yet conducted – trial completed at Phase 1, none ongoing
	Emibetuzumab/LY2875358/LA480	Not yet conducted – trials completed and ongoing at Phase 2
	Amivantamab/JNJ-61186372/JNJ-372	NCT04487080
	ABT-700/h224G11	Not yet conducted – trials completed at Phase 1 and ongoing at Phase 2
MET kinase inhibitors	Tivantinib/ARQ 197	NCT02029157, NCT01755767, NCT01244191
	Savolitinib/AZD6094/HMPL-504/HMP-504/Volitinib	NCT03091192
	Tepotinib/MSC2156119J/EMD1214063	Not yet conducted – trials completed at Phase 1 and ongoing at Phase 2
	Glesatinib/MGCD265	Not yet conducted – trials completed and ongoing at Phase 2
	Capmatinib/INC280	NCT04427072, NCT03784014
	PHA665752	Not yet conducted – no trials completed at any Phase
	SU11274	Not yet conducted – no trials completed at any Phase
	Foretinib/GSK1363089/ XL880	Not yet conducted – trials completed and ongoing at Phase 2
	Merestinib/LY2801653	Not yet conducted – trials completed at Phase 1 and ongoing at Phase 2
	MK8033	Not yet conducted – trial completed at Phase 1, none ongoing
Multi-kinase inhibitors	Crizotinib/PF-02341066 *Targets: MET, ALK, ROS, RON*	NCT02838420, NCT02075840, NCT01639001, NCT00932893, NCT01154140, NCT04009317, NCT03052608, NCT02737501, NCT02767804, NCT02201992, NCT03194893, NCT03126916, NCT03596866, NCT03874273
	Cabozantinib/XL184, BMS907351 *Targets: MET, VEGFR2, KIT, RET, AXL, FLT3*	NCT01908426, NCT01605227, NCT01865747, NCT00704730, NCT03690388, NCT04338269, NCT03755791, NCT03937219, NCT03375320, NCT04446117, NCT03793166, NCT03729245, NCT03141177, NCT04211337
	Amuvatinib/MP470 *Targets: MET, c-Kit, PDGFRα, Flt3, c-Ret*	Not yet conducted – trial completed at Phase 2, none ongoing

As our understanding of the molecular biology of cancer has progressed, drug development has shifted towards a more targeted and personalised approach to treatment. However, despite a growing number of targeted cancer therapies, cytotoxic chemotherapy remains the mainstay of treatment for malignant disease. Different classes of chemotherapy and their clinical uses are outlined in Table 2. Most agents are directed against the processes of DNA replication and mitosis, utilising malignant cells’ defective DNA repair pathways and triggering cell death [18]. Some cancer types, such as glioblastoma and pancreatic cancer, are known to be innately chemoresistant and are characterised by a lack of response to initial chemotherapy. The majority of tumours develop acquired chemotherapy resistance, where an initially chemosensitive tumour adapts in response to chemotherapy through various mechanisms including: (i) increased cell viability which can be directly triggered by impaired apoptosis or increased proliferation [19], (ii) improved DNA repair [19], (iii) increased drug efflux and altered drug metabolism [19–21], (iv) propagation of cancer stem cells [22], (v) increased invasive potential [23] and (vi) increased tumour hypoxia with altered angiogenesis [24]. All of these mechanisms can lead to increased cancer cell viability. Often, a combination of these mechanisms is observed in chemoresistant cells.

**Table 2:** An overview of chemotherapeutic agents, their mechanism of action and indication.

Class of chemotherapy	Subtype	Drugs	Mechanism of action	Indication
Alkylating agents	Nitrogen mustards	Cyclophosphamide, Melphalan, Ifosfamide, Chlorambucil, Busulfan	Alkylate proteins, RNA and DNA. Covalently bind to DNA via alkyl group causing intra and interstrand cross-links	Multiple myeloma, sarcoma, breast cancer, lymphoma
	Nitrosoureas	Carmustine, Lomustine, Stretozotocin		Glioblastoma multiforme
	Tetrazines	Dacarbazine, Temozolamide		Glioblastoma multiforme, Hodgkin’s lymphoma
	Aziridines	Mytomycin		Gastric cancer
	Platinums	Cisplatin, Carboplatin, Oxaliplatin		Ovarian, gastric, lung, head and neck cancer, sarcoma
Antimetabolites	Anti-folates	Methotrexate, Pemetrexed	Inhibit enzymes essential for thymidylate and purine production	Lung cancer, sarcoma
Antimicrotubule agents	Vinca alkaloids	Vincristine, Vinblastine, Vinorelbine	Bind to tubulin inhibiting assembly into microtubules	Lung cancer
	Taxanes	Docetaxel, Paclitaxel	Promote microtubule stability, preventing disassembly	Breast, ovarian cancer
Topoisomerase inhibitors	Topoisomerase I inhibitors	Irinotecan, Topotecan, Camptothecin	Block unwinding of the DNA double strand helix and prevent DNA synthesis and translation	Lung cancer, colorectal cancer
	Topoisomerase II inhibitors	Etoposide, Doxorubicin		Lung cancer, sarcoma
Cytotoxic antibiotics	Anthracyclines	Doxorubicin, Epirubicin	Intercalates DNA, topoisomerase II inhibitor, generate reactive oxygen species (ROS), DNA adduct formation	Breast cancer, oesophageal cancer
	Bleomycins	Bleomycin		Hodgkin’s lymphoma, testicular cancer

It is known that MET confers resistance to targeted agents including BRAF inhibitors in malignant melanoma and the EGFR inhibitor gefitinib in NSCLC, through activating oncogenic signalling pathways such as PI3K/AKT [25–27]. This review compiles the recent evidence that MET also promotes resistance to chemotherapeutic agents. Furthermore, MET inhibition reduces the viability of chemoresistant cancer cells and can synergise with chemotherapy. We also explore the molecular mechanisms that could be exploited to overcome MET-driven chemotherapy resistance.

## Role of MET in chemoresistance and mechanisms involved

**Fig. 2:**
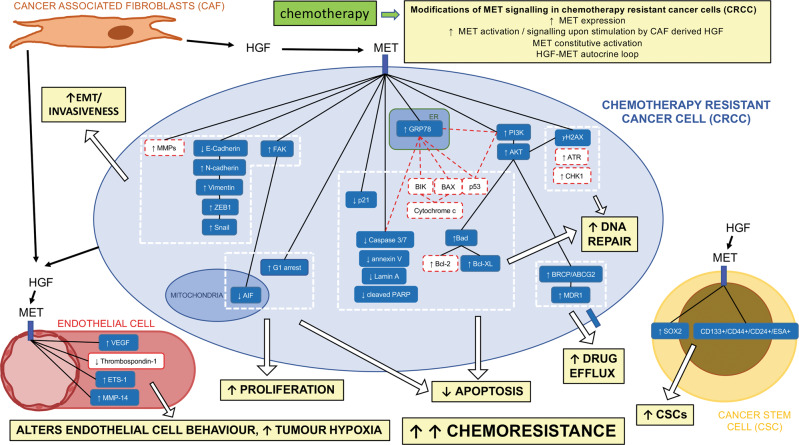
Model of MET-driven chemoresistance. Chemotherapy resistant cancer cells (CRCC) have altered MET signalling, including overexpression of MET, activation sustained by HGF secreted by CAFs (cancer associated fibroblasts), constitutive activation of MET and secretion of HGF which is not normally expressed by epithelial cells, leading to an autocrine activation loop. This diagram illustrates the effect of this altered MET signalling on: (i) the behaviour of CRCCs: reduction of apoptosis, increased proliferation, enhanced DNA repair, upregulation of drug efflux and stimulation of epithelial-mesenchymal transition; (ii) changes in the tumour microenvironment: alteration of the behaviour of endothelial cells and increased intra-tumoural hypoxia, promotion of cancer stem cells (CSCs) survival and proliferation. All these changes contribute to the development of chemotherapy resistance. The intracellular pathways reported or suggested are shown. Blue box/black line: confirmed mechanism. Dotted red box/red line: proposed mechanism. MMPs matrix metallopeptidases, VEGF vascular endothelial growth factor, AIF apoptosis-inducing factor, ER endoplasmic reticulum.

Recent preclinical studies have shown that MET is involved in the above-cited key mechanisms of chemoresistance, and point towards a synergy between chemotherapy and MET inhibition. The studies use distinct cell lines with differing degrees of chemosensitivity or isogenic pairs of parental and chemoresistant cells. Several studies demonstrate that HGF reduces cell sensitivity to chemotherapy. Others report enhanced MET signalling in the chemoresistant cells compared to the chemosensitive or less resistant cells. This enhanced MET signalling results from: (i) overexpression of MET [28–36], (ii) constitutive activation of MET [28, 29, 37], (iii) activation sustained by HGF [30, 37], and (iv) secretion of HGF, not normally expressed by epithelial cells, leading to an autocrine activation loop [28, 33, 34, 37]. Interestingly, a number of studies also found HGF to already be secreted by chemosensitive cells [28, 29, 32, 37–40]. It is possible that this pre-existing HGF plays a role in the acquisition of chemoresistance, although this remains to be determined. We describe below the results of these studies. The following experimental details from each study are presented in Table 3: cancer type, chemotherapy agent tested, cell models used (divided by chemosensitive and chemoresistant cell lines), method of MET/HGF blockade employed, observed changes in MET/HGF leading to enhanced MET signalling and the MET-dependent mechanisms of chemotherapy resistance. Figure 2 illustrates the mechanisms of MET-driven chemoresistance reported in these studies.

**Table 3:** Table of all the preclinical studies evidencing the role of MET in chemoresistance and the mechanisms involved.

Cell lines in blue font: paired isogenic chemosensitive and chemoresistant cell lines. (Note - some cell lines placed in the chemosensitive category in this table have also been used as a model to study chemoresistance as their chemosensitivity may be reduced). Red box: MET overexpressed in chemoresistant cell line compared to parental cells. Yellow box: constitutive activation of MET. Orange box: MET overexpressed and constitutively active. Green dot: HGF expressed. The darker or lighter green indicate an increase or a decrease of HGF in chemoresistant cells versus chemosensitive cells.

MET-stimulated cellular functions which promote chemoresistance: A: apoptosis inhibition, R: DNA repair enhancement, CC: cell cycle progression, D: drug efflux, P: increased proliferation, E: increased EMT, S: enhanced cancer stem cells survival and proliferation, AE: altered endothelial cell behaviour, H: increased intra-tumoural hypoxia.

*NSCLC* non-small cell lung cancer, *SCLC* small cell lung cancer, *PDAC* pancreatic ductal adenocarcinoma, *HCC* hepatocellular carcinoma.

### MET increases cell viability through reducing apoptosis and/or increasing proliferation in chemoresistant cells

Multiple studies report that the observed enhanced MET signalling occurring in chemoresistant cells increases their viability. This occurs through a decrease in apoptosis, as shown in most studies, but also through stimulation of proliferation. Proliferative activity is a vital component of cancer development and progression, with the MET/HGF axis being a known molecular regulator. Apoptosis, the principal route of cell death triggered in response to chemotherapy, is regulated by a multitude of different factors. One important group is the Bcl family of proteins, including Bcl-2, Bcl-XL and Bad. MET has been shown to influence the regulation of pro-apoptotic and anti-apoptotic factors to promote cell survival or proliferation through activating many signalling pathways including the Mitogen-Activated Protein Kinase (MAPK) cascade such as ERK1/2, Jun amino-terminal kinases (JNKs), p38, PI3K-AKT and STAT3 [41, 42]. On phosphorylation, AKT promotes cell survival pathways mediated by Bcl-2 and Bcl-XL [43].

Several studies have reported the protective effect of HGF against chemotherapy-induced cell death or the effect of MET inhibition/loss of expression in reducing chemoresistance.

#### HGF decreases cells’ sensitivity to chemotherapy through reducing apoptosis

Exogenous HGF [39, 44–48] or endogenous HGF present in the conditioned media of cancer associated fibroblasts (CAFs) [38], has been shown to protect breast, glioblastoma, lung, ovarian cancer and osteosarcoma cells against apoptosis induced by the DNA damaging agents doxorubicin, cisplatin, camptothecin or paclitaxel.

The mechanisms reported were: (i) prevention of the downregulation of Bcl-X_L_; [44, 47] (ii) activation of ERK1/2 and the PI3K/AKT-dependent anti-apoptotic pathway; [38, 39, 48] (iii) activation of focal adhesion kinase (FAK) [46] and (v) reduction of apoptosis-inducing factor (AIF) expression [46]. AIF is a protein located within the mitochondria, whose activation leads to a caspase-independent pathway of apoptosis by mediating DNA fragmentation and chromatin condensation [49]. Another reported mechanism is the induction of expression of the molecular chaperone protein GRP78 (BiP) [38]. BiP has been reported to inhibit apoptosis by interactions with caspase-7 or p53 [28, 38], and through preventing the release of cytochrome c by binding to BIK and BAX [50, 51].

Interestingly, the expression of HGF (through HGF cDNA transfection) in Chinese hamster ovary cells reduced their sensitivity to chemotherapy [52]. Moreover, an autocrine loop of MET activation has been reported in several chemoresistant cancer cell lines, leading to their increased viability [2, 28, 37]. For example, Lasagna et al. reported that a multi-drug resistant (MDR) hepatocellular carcinoma (HCC) cell line secreted higher levels of HGF than the isogenic parental cell line, enhancing their proliferative activity [34].

#### Chemoresistant cells display enhanced MET signalling and MET-targeted therapy reduces their survival by increasing apoptosis and/or decreasing proliferation

Several studies have shown that MET expression and/or activation is increased in cells with acquired chemoresistance compared to their isogenic parental cell lines. This in turn leads to an increase in MET signalling with enhanced sensitivity to MET inhibition and reversal of chemoresistance.

For example, cisplatin-resistant ovarian cancer cell lines were found to express high levels of MET compared to parental chemosensitive cells. On treatment with cisplatin, MET phosphorylation was increased, but upon MET inhibition with PHA-665752 or *MET* siRNA knockdown cisplatin-induced apoptosis was enhanced [30]. In a similar way, treatment of osteosarcoma cell lines with PHA-665752 or with a neutralising anti-HGF antibody enhanced the cytotoxic effect of cisplatin [45].

Small cell lung cancer (SCLC) cells with acquired chemoresistance to paclitaxel, cisplatin or SN38 [28], MDR multiple myeloma cell lines [29], MDR uterine sarcoma and breast cancer cell lines (that overexpress ABCB1/MDR1) [33] were found to display higher levels of MET protein expression and phosphorylation than their respective isogenic parental cells. The viability of chemoresistant cells was significantly reduced upon treatment with the MET inhibitor SU11274 [28, 29], *MET* siRNA [28] or *MET* shRNA [33] knockdown. This occurred through reduced proliferation [29] or enhanced apoptosis [28, 29, 33] as evidenced by the detection of cleaved PARP [28, 33] or increased caspase 3/7 activity and annexin V staining [33]. The *MET* gene was found to be amplified in chemoresistant SCLC cells [28], which could explain MET protein overexpression. Some studies have found that chemoresistant cells express higher levels of HGF versus their isogenic chemosensitive parental cells which likely explains the basal MET phosphorylation [28, 33]. This indicates a switch to autocrine MET activation, which could play a major role in the acquisition of chemotherapy resistance. Patient-derived multiple myeloma plasma cells were found to have higher MET expression and basal phosphorylated MET in patients at disease relapse compared to those at diagnosis or in remission. SU11274 reduced the viability of plasma cells from relapsed, chemoresistant patients [29]. Interestingly, the growth of tumour xenografts formed by ABCB1/MDR1-overexpressed MDR uterine sarcoma and breast cancer cell lines, knocked down for *MET* with shRNA, was reduced compared to tumours formed by control shRNA transfected cells [33].

These studies demonstrate that MET signalling is enhanced in various chemoresistant cell lines and that MET inhibition can reverse chemotherapy resistance.

#### Pharmacological inhibition or genetic silencing of MET synergises with chemotherapy through reducing apoptosis

A number of preclinical studies have demonstrated that MET inhibition is able to reverse chemotherapy resistance in various different tumour types.

A synergy between MET inhibition (using MK8033 [53], capmatinib [54], crizotinib [55], PHA-665752 [30, 45, 56] or SU11274 [29]) and chemotherapy (carboplatin [53], paclitaxel [53, 54], cisplatin [30, 45, 55], doxorubicin [29, 56] or bortezomib [29]) has been reported in ovarian [53–55], gastric [56], osteosarcoma [45] and MDR multiple myeloma [29] cancer cell lines. Many of these cell lines were found to exhibit constitutive MET activation [29, 30, 54, 56] and increased MET expression [29]. Cell viability was reduced due to apoptosis induction, evidenced by upregulation of the enzymatic activity of caspase-3 and of the expression of cleaved caspase-3 and cleaved lamin A [30, 54–56]. Such synergy was also observed in vivo, with crizotinib and cisplatin combination treatment reducing ovarian tumour growth more than either therapy alone [55]. Interestingly, in several ovarian cancer cell lines, enhanced sensitivity to carboplatin and paclitaxel with MET inhibition (MK8033) has been correlated with a 47 gene signature. Furthermore, the expression of these genes in patient samples was associated with overall survival [53], suggesting that this signature may be utilised as a prognostic biomarker.

In the genetic PDAC mouse model KPC (K-ras^LSL.G12D/+^; p53^R172H/+^; PdxCre mice) and orthotopic transplantation of KPC derived cells, MET inhibition with capmatinib sensitised PDAC tumours to gemcitabine, resulting in a reduction in primary tumour volume and metastatic burden via increased apoptosis [57].

In addition to pharmacological inhibition, genetic silencing of *MET* has been shown to reverse chemoresistance in different tumour types. In several glioma cell lines [58, 59] and in a multiple myeloma cell line [60], the reduction of MET expression by shRNA-mediated knockdown [58, 60] or antisense oligodeoxynucleotides [59] enhanced the effect of temozolamide [58], paclitaxel [59] or doxorubicin [60], in reducing cell survival. This was proposed to be via increased apoptosis with a higher apoptotic cell count [59] or higher levels of cleaved caspase 3 fragments and cleaved PARP [60] with the combination treatment compared to either monotherapy. Moreover, low MET expression in glioma patient samples was shown to be associated with enhanced response to alkylating chemotherapy agents and prolonged overall survival [58].

Exosome-delivered MET-specific siRNA (exo-si-c-Met) reversed resistance to cisplatin in gastric cancer cells in vivo and in vitro [61]. In vitro, exo-si-c-Met was shown to promote apoptosis in chemoresistant but not isogenic chemosensitive cells. In vivo, exo-si-c-Met combined with cisplatin treatment had a synergistic effect, reducing growth of chemoresistant tumours [61].

These studies highlight the importance of pursuing combination therapy with MET inhibition and chemotherapy in clinical trials.

### MET enhances DNA repair via promoting the chemotherapy-induced DNA damage response

DNA damage can be caused by exogenous and endogenous sources. Endogenous examples include the production of reactive oxygen species (ROS), produced as a natural by-product of normal metabolism, whilst exogenous sources include ionising radiation and chemotherapy. The pathways employed in DNA repair include base excision repair (BER), nucleotide excision repair (NER), mismatch repair (MMR), homologous recombination (HR) and non-homologous end-joining (NHEJ). These are commonly dysregulated in cancer in order to facilitate tumorigenesis [62]. The DNA damage response is activated by three main kinases, ATM, ATR and DNA-PK [63].

Studies have shown that repair of chemotherapy-induced DNA damage can be inhibited by MET activation or inhibition. HGF stimulation of breast and prostate cancer cells was shown to enhance the rate of repair of DNA double strand breaks (DSBs) in response to doxorubicin [47]. Interestingly, Bcl-XL, whose downregulation was blocked by HGF, was shown to enhance DNA repair, although it is normally recognised as an anti-apoptotic protein [47].

Conversely, in ovarian [54] and gastric [56] cancer cell lines that display basal MET phosphorylation, MET inhibition with capmatinib [54] or PHA-665752 [56], in combination with paclitaxel [54] or adriamycin [56], has been shown to lead to accumulation of DSBs compared to chemotherapy alone. This was demonstrated by increased levels of the DNA damage response protein γH2AX [54, 56]. As well as increasing γH2AX levels, PHA665752 was shown to trigger γH2AX tyrosine phosphorylation and its subsequent association with pro-apoptotic kinase JNK1 [56]. γH2AX tyrosine phosphorylation has also been shown to hinder histones’ capacity to interact with DNA repair effectors following DSBs. Destabilisation of the ATR-CHK1-CDC25B DNA damage response pathway was also detected with PHA-665752, with decreased levels of phosphorylated ATR and CHK1 [56]. This would allow cells with damaged DNA to progress through the cell cycle, at which point DSBs and detrimental chromosomal aberrations would trigger apoptosis.

These studies indicate that MET is able to protect cancer cells against the chemotherapy-induced DNA damage response and hence MET inhibition can suppress DNA repair.

### MET upregulates drug efflux

ATP-binding cassette (ABC) transporters are crucial transport proteins which regulate the removal of substances from the cell through the plasma membrane in an ATP-dependent process [64]. Altered drug efflux is a well-recognised mechanism of drug resistance in cancer. For example, overexpression of the BRCP/ABCG2 transporter is associated with resistance to several anti-cancer drugs including doxorubicin, mitoxantrone and topotecan [65], whilst ABCB1/MDR1 is frequently overexpressed in drug-resistant cell lines and is found to correlate with poor overall survival in patients [20, 66].

MET can upregulate the expression of efflux transporters in the cell membrane, thus reducing the intracellular concentration of the chemotherapeutic agent. MET overexpression was shown to trigger an increase of BRCP/ABCG2 and ABCB1/MDR1 expression in doxorubicin-resistant (DR) ovarian carcinoma cells [32] and in cisplatin-resistant NSCLC cell lines [31] respectively. Consistent with these findings, MET and ABCB1/MDR1 were overexpressed in MDR uterine sarcoma and breast cancer cell lines, compared to their isogenic parental cell lines [33]. It is thought that MET controls BRCP/ABCG2 at the transcription level via PI3K/AKT activation [32]. MET inhibition with SU11274 and silencing *MET* by shRNA repressed BRCP/ABCG2 [32] and ABCB1/MDR1, leading to enhanced chemosensitivity [31]. SU11274 was also reported to act in synergy with SN38 in reducing the expression of BRCP/ABCG2 in gastric cancer stem cells [67].

Thus MET overexpression can promote an increase in drug efflux through regulating the expression of efflux transporters, leading to chemoresistance and increased cell survival.

### MET promotes the survival and proliferation of chemoresistant cancer stem cells

Cancer stem cells (CSCs) are thought to generate differentiated tumour cells, which have limited proliferative potential, and are responsible for seeding tumour populations [68]. CSCs have been heavily implicated in mediating chemoresistance in many cancers, including glioblastomas, pancreatic and colorectal cancers [22]. This is thought to be secondary to the cells’ ability to lie dormant, increase DNA repair and drug efflux capacity, and decrease apoptosis. Thus, CSCs persist through chemotherapy treatment and facilitate relapse through repopulation after treatment has been discontinued. CSCs are characterised differently depending on their tissue of origin, but some common markers used to identify them include ESA, CD44, CD24 and CD133 [69].

In a number of human tumours, overexpression of MET leads to the acquisition of a stem cell-like phenotype [70, 71]. It is thought that overexpression of MET may facilitate the formation of CSCs from normal stem cells in the tumour microenvironment, or alternatively may encourage the de-differentiation of mature cells [36]. As a result, MET may be pivotal in the initial development of a tumour, as well as in its progression and ability to persist through cytotoxic treatment.

Hage et al. demonstrated that the MET inhibitor cabozantinib increased gemcitabine efficacy through triggering apoptosis and reducing the expression of several CSC markers, including SOX2 and CD133 in pancreatic ductal adenocarcinoma (PDAC) cells [72]. Li et al. reported that a proportion of primary PDAC CSCs express high levels of MET and have a tumourigenic phenotype in vitro and in vivo. The MET inhibitor cabozantinib and *MET* shRNA knockdown significantly reduced their tumourigenicity in vitro and in vivo and reduced the population of CSCs. This demonstrates the role of MET in CSCs’ tumourigenicity. Moreover, combination treatment with cabozantinib and gemcitabine inhibited tumour growth and metastatic spread more than either drug alone [73]. Consistent with this, Yashiro et al. reported a synergistic anti-proliferative and pro-apoptotic effect of the MET inhibitor SU11274 with SN38 in chemoresistant CSCs derived from gastric cancer cell lines (side population-enriched cancer stem cells (SP-CSCs)) [67]. It was noted that a smaller proportion of SP-CSCs than the parental cells were in the replication (S) phase of the cell cycle. Treatment with SU11274 increased the number of chemoresistant cells in S-phase. As SN38 is specifically active in S phase, this further explains the synergistic effect seen with combination treatment. SU11274 and SN38 also synergistically reduced the growth of tumour formed by the chemoresistant CSCs in mice [67].

These studies indicate that MET is a key driver of cancer cell stemness that leads to chemoresistance.

### MET may lead to chemoresistance through stimulating epithelial-mesenchymal transition

The process of epithelial-mesenchymal transition (EMT) enables cells to invade surrounding tissues and metastasise, with enhanced migratory capacity, invasiveness, production of extracellular matrix components and resistance to apoptosis [74, 75]. EMT has been shown to contribute to chemoresistance [23, 76]. Upon activation, MET is known to drive cancer cell invasion and metastasis through altering cell-cell and cell-surface adhesions and the actin cytoskeleton, leading to the acquisition of cell motility, including the formation of invadopodia [77]. MET does this by upregulating the expression of EMT markers such as vimentin and Snail and the expression or activity of matrix metalloproteinases (MMPs), which digest the surrounding stroma to promote invasion [78–80].

Culturing a SCLC cell line in the presence of HGF for 10–14 days induced EMT features, with increased Snail expression and invasiveness. The mesenchymal subpopulation of this cell line was found to secrete HGF, thus leading to an autocrine loop of MET activation. These mesenchymal cells displayed an HGF/MET-dependent EMT phenotype and acquired etoposide resistance both in vitro and in vivo xenograft experiments in nude mice. This chemotherapy resistance was reversed upon MET inhibition using crizotinib. Interestingly, an upregulation of phosphorylated MET and mesenchymal markers were observed in SCLC patient samples at relapse [37].

Two cisplatin-resistant NSCLC cell lines display MET overexpression and increased expression of N-cadherin, vimentin, ZEB1 and Snail and reduced expression of E-cadherin compared to their isogenic parental cells [31]. MET, through the PI3K/AKT/mTOR signalling pathway was found to promote the acquisition of this EMT phenotype. Thus, overexpression of the microRNA miR-206, or knockdown of its target MET, reversed these mesenchymal features and sensitised resistant cells to cisplatin [31].

These studies suggest that MET-dependent EMT confers chemoresistance although a direct link remains to be clearly demonstrated.

### MET alters endothelial cells’ behaviour and promotes tumour hypoxia

During tumorigenesis cancers outgrow their blood supply and develop a necrotic, hypoxic core. Hypoxia facilitates cell survival under stress, by causing cell cycle arrest and downregulation of apoptosis, senescence and mitochondrial activity [81]. Hypoxia also induces cellular adaptations which can hinder the efficacy of chemotherapy, such as reduced cellular uptake of drugs due to increased cellular acidity and drug efflux pump expression [82], whilst some chemotherapeutic agents require oxygen in order to facilitate their cytotoxicity [24].

In response to intra-tumoural hypoxia, cancer cells express hypoxia-inducible factor-1α (HIF-1α), which induces the expression of the pro-angiogenic vascular endothelial growth factor (VEGF), MET and HGF. The MET/HGF signalling axis itself promotes cancer cells’ upregulation of VEGF and downregulates the expression of anti-angiogenic proteins, such as thrombospondin 1. In turn, VEGF or HGF can bind on VEGFR or MET, both expressed on the surface of endothelial cells, stimulating their proliferation, migration and angiogenesis [83].

Two different relationships between chemoresistance and MET activated on endothelial cells through cancer cell-derived HGF have been shown.

Proliferation, survival, migration, tubulogenesis and in vivo neovascularisation of HUVEC (human umbilical vein endothelial cells) was increased significantly more with the HGF-containing conditioned medium of MDR HCC cells, compared to the conditioned medium of the isogenic parental chemosensitive cells, which contained no HGF [34]. Furthermore, siRNA knockdown of the multi-resistance gene MDR1 reduced HGF production from MDR HCC cells [34].

Huang et al. reported that the HGF-containing conditioned medium of patient-derived glioblastoma cancer cells drives endothelial cells to acquire a fibroblastic phenotype, in a process called endothelial-mesenchymal transition (Endo-MT) [40], resulting in abnormal angiogenesis, vessel leakage and hypoxia. In vivo endothelial cell-specific knockout of *MET* (through the generation of Tie-Cre Met^fl/fl^ mice) led to normalised vasculature, reduced intra-tumoural hypoxia, slowed glioblastoma growth and importantly, sensitised glioblastoma tumours to temozolomide treatment, prolonging mouse survival [40].

These two studies indicate that MET activation on endothelial cells by HGF secreted by cancer cells can facilitate chemoresistance in two different ways. Drug resistance can trigger an increased production of HGF by cancer cells, leading to tumour angiogenesis. Alternatively, HGF secretion by cancer cells can trigger chemoresistance through altering angiogenesis and triggering hypoxia.

## Clinical uses of MET inhibitors in chemoresistance

As discussed above, a number of clinical trials have evaluated MET inhibitors as a monotherapy against other targeted therapies or standard of care chemotherapy regimens (see Table 1). Crizotinib and cabozantinib were approved based on superiority to chemotherapy and the mTOR inhibitor everolimus, respectively [84, 85]. These two drugs are multi-kinase inhibitors, however, so it is difficult to draw conclusions about whether the positive results were associated with their MET activity. Capmatinib is a selective MET inhibitor that has been granted accelerated FDA approval for patients with NSCLC whose tumours harbour *MET* exon 14 mutation [16]. None of these trials directly addressed the reversal of chemotherapy resistance with MET inhibition.

Following strong preclinical evidence for the additive or synergistic efficacy of MET inhibitors in combination with chemotherapy, a number of clinical trials assessing combination therapy have been conducted in solid cancers. These trials are outlined in Table 4. Trial data is mature in gastric, prostate and colorectal cancers. Unfortunately, no trial to date has demonstrated a significant improvement in median overall survival (mOS) with the addition of MET-targeted therapy to chemotherapy. The phase II trial investigating the addition of the anti-HGF antibody rilotumumab to ECX (epirubicin, cisplatin and capecitabine) chemotherapy in gastric adenocarcinoma demonstrated a modest improvement in progression-free survival (PFS, 5.7 vs 4.2 months) and objective response rate (ORR, 39% vs 21%) [86] compared to placebo plus ECX. However, the phase III trial of this combination had to be terminated early due to fatal side effects (including febrile neutropenia) in the rilotumumab arm [87]. In the phase II study, the MET positive subgroup of patients, as determined by IHC, had a prolonged mOS in the rilotumumab arm compared to the placebo arm [86]. In other trials, however, no correlation between MET expression and response to MET-targeted therapy was observed.

**Table 4:** A summary of the clinical trial results of MET inhibitors alongside chemotherapy versus chemotherapy alone.

This table summarises the results that are currently available for completed Phase II/III clinical trials using MET/HGF inhibitors alongside chemotherapy.

*N* sample size, *ORS* objective response rate (%), *PFS* progression free survival, *mOS* median overall survival, *ECX* epirubicin, cisplatin and capecitabine, *IHC* immunohistochemistry, *CRPC* castration-resistant prostate cancer, *CRC* colorectal cancer, *mFOLFOX6* fluorouracil, leucovorin and oxaliplatin.

The red box indicates that there was a correlation between MET expression and the study outcome.

^a^0 (unstained), 1+ (weak staining), 2+ (moderate staining), and 3+ (strong staining).

^b^*p*-values not reported.

## Optimising preclinical research and future directions for the clinical utility of MET inhibitors in chemoresistant disease

The modest clinical benefit of MET/HGF inhibitors in combination with chemotherapy in clinical trials necessitates review of why the preclinical evidence for these compounds is not translating into clinical practice.

### The use of appropriate modelling systems

In this review we have outlined the cell-based models used to demonstrate the mechanisms of MET-driven chemoresistance. It is important to recognise the limitations of using cell lines in research. For example, Domcke et al. recently highlighted that a number of the more commonly used cell lines in preclinical ovarian cancer research do not accurately represent human disease [88]. A number of studies that we have discussed used cell lines of tumour types that are known to be chemoresistant, such as glioblastoma, whilst other groups created novel chemotherapy resistant cell lines by culturing cells in increasing doses of cytotoxic chemotherapy. Isogenic chemosensitive and resistant cell pairs enabled a direct comparison between chemoresistant and chemosensitive cells lines, and highlighted aberration of the MET/HGF pathway as a common feature of chemoresistant cell lines (see Table 3). However, one possible limitation to such otherwise powerful models is that they may not accurately reflect the chemoresistance seen in human disease. Therefore, results obtained in isogenic models would highly benefit from validation in patient-derived primary cells and human samples in future studies.

2D models have formed the basis of many scientific discoveries, but the use of 2D models can result in cellular behaviour that deviates dramatically from the in vivo response. In addition, many important characteristics of cancer cells cannot be appropriately modelled in 2D cultures, including exposure to growth factors, cellular polarity and cellular adhesion [89, 90]. The majority of preclinical studies investigating the efficacy of MET inhibitors in vitro use 2D models, such as clonogenic assays and cell viability assays. Indeed, a review of the preclinical evidence for MET inhibitors in pancreatic cancer acknowledged that none of the preclinical cancer models had considered the role of the stroma in MET/HGF pathway signalling [91]. In pancreatic cancer, the predominant source of HGF is from pancreatic stellate cells (PSCs), but many of the in vitro studies only included endothelial cells and vascular smooth muscle cells to represent the stroma [92].

In recent years, a number of novel 3D cell culture models have been developed to better replicate the dimensionality of the extracellular matrix. As a result, 3D models have proven to be more capable of inducing in vivo-like cell fates than their 2D counterparts. Expanding the use of 3D models in MET-targeted preclinical research is therefore likely to provide data that will better inform in vivo and clinical testing.

A major proportion of in vivo research on MET inhibitors has been performed in immunosuppressed mice to allow for xenotransplantation of human cells without rejection. There is increasing evidence that the tumour microenvironment (TME) that comprises cancer cells, tumour stroma, blood vessels and immune infiltrate, promotes cancer progression and alters tumour sensitivity to therapy. Until now the interplay between MET signalling and the immune system has been largely uncharacterised. One key study has demonstrated that MET/HGF signalling is involved in mediating the recruitment of neutrophils into the TME, and as such is thought to play a role in regulating tumour immunity to some therapies [93]. Finiguerra et al. similarly showed that MET is required for neutrophil chemoattraction and cytotoxicity in response to HGF; however, they also demonstrated that the effect of MET tyrosine kinase inhibitors was tempered by a pro-tumoural effect of MET blockade in neutrophils [94]. In light of this, the use of immunocompetent mice models, such as genetically engineered (GEM) or syngeneically transplanted models would enhance the ability to effectively represent MET-mediated cellular behaviours. This would provide a more accurate depiction of the action of MET inhibitors in vivo, and subsequently their likely clinical benefit.

### Improved biomarkers for tumour/patient selection

Accurate patient selection for MET-targeted therapy is vital. A number of studies have determined that increased expression of MET is linked with poor clinical outcomes. For example, MET overexpression has been associated with more invasive advanced tumours in glioblastoma [95] and oesophageal squamous cell carcinoma [96], a significantly increased risk of tumour recurrence in colorectal cancer [97], and reduced survival in glioblastoma [58, 95], colorectal cancer [97] and breast cancer [98, 99]. Clinicopathologic associations have also indicated that MET overexpression is associated with resistance to chemotherapy in breast cancer [98, 99] and oesophageal squamous cell carcinoma [96].

The modest effect of MET-targeted treatments seen in clinical trials to date may be due to inadequate patient stratification. Patient stratification has unfortunately been hindered by the lack of a reliable biomarker of MET activity. As seen in Table 4, all of the clinical trials testing MET/HGF-directed therapy alongside chemotherapy assessed MET expression using immunohistochemical (IHC) staining of tumour samples. Thus, tumours were stratified by total MET cell surface expression. With the exception of the phase II study of the anti-HGF antibody rilotumumab in gastric cancer, all trials demonstrated no relationship between MET expression and response to MET inhibitors [100].

The use of total MET as a surrogate marker for pathway activation is questionable for the following reasons:

(i) Levels of expression do not necessarily correlate with levels of activity. For example, MET may still be highly activated in tumours with low expression if the level of HGF in the TME is high. Also, oncogenic mutations that trigger MET activation do not necessarily trigger MET overexpression [101].

(ii) As discussed above, total MET IHC is usually scored on staining within the plasma membrane. MET has been shown to remain active post-endocytosis and furthermore to require endocytosis to sustain its activation and/or downstream signalling [3–5]. Therefore, new scoring methods that take into account MET staining in the cytoplasm, in addition to the plasma membrane, will enhance patient selection and subsequent outcomes.

(iii) Importantly, in all preclinical and clinical studies to date, different antibodies, molecular tests and evaluation criteria have been used, limiting the validity of the outcomes. Unlike other molecular markers, such as the oestrogen receptor and HER2, robust comparable testing and evaluation criteria for MET have not yet been developed.

Many trials performed exploratory analyses of other potential biomarkers such as serum HGF level, tumour HGF level, *MET* gene copy number (amplification) and soluble MET concentrations (see Table 4). Unfortunately, no clinically meaningful correlation was seen between any of these biomarkers and treatment response. Consistent with this, one study reported that serum MET levels were not indicative of MET present within tumours [102]. This relationship has also been explored using cell line panels and a lack of correlation between mRNA expression, protein expression and activation of MET was seen [103].

The only indicators proven to correspond to pathway activity are MET phosphorylation (phospho-MET) and *MET* exon 14 deletions [104], but *MET* mutations are rare in general cancer populations. Phospho-MET has not been used as a selection criterion for clinical trials so far [103]. Unfortunately, there are significant limitations with phospho-MET antibodies, as they have poor sensitivity and specificity [17, 37]. In addition, there are conflicting reports about the correlation between MET expression and phosphorylated MET on patient samples. One study has shown no link between the staining patterns [105], whereas another noted that MET phosphorylation is associated with ‘high’ MET scores. Copin et al. demonstrated that in NSCLC tumours phosphorylated MET was restricted to <20% of the tumour cells and mostly at the invasive front [102]. As a consequence, and also for the reasons stated above, the levels of activity do not necessarily correlate with level of expression.

Therefore, until a reliable biomarker of MET activity is elucidated, the method of patient stratification in trials will continue to be a matter for debate. It is possible that once biomarkers used for selection of patients for targeted therapies against MET have been adequately established, they might also be used for as an adjunct for identification of resistance to chemotherapy.

## Conclusion

In conclusion, chemotherapy resistance remains one of the fundamental barriers to effectively treating cancer. MET provides a promising target to reverse chemoresistance, but to date there has been poor translation of preclinical data to positive clinical trials outcomes. By further exploiting the mechanisms of MET-mediated chemoresistance and optimising patient stratification, there is hope for improved outcomes in targeting MET in patients with chemotherapy resistant cancers.
